# Comparative Analysis of Right vs. Left Radial Access in Percutaneous Coronary Intervention: Impact on Silent Cerebral Ischemia

**DOI:** 10.3390/medicina60081193

**Published:** 2024-07-23

**Authors:** Abdulkadir Kara, Korhan Soylu, Ufuk Yildirim, Muhammet Uyanik, Metin Coksevim, Bahattin Avci

**Affiliations:** 1 Department of Cardiology, Elbistan State Hospital, 46300 Kahramanmaraş, Turkey; 2 Department of Cardiology, Faculty of Medicine, Ondokuz Mayis University, 55270 Samsun, Turkey; korhansoyly@yahoo.com (K.S.); ufukyildirim2715@yahoo.com (U.Y.); metincoksevim@gmail.com (M.C.); 3 Department of Cardiology, Carsamba State Hospital, 55500 Samsun, Turkey; muhammetuyanik@hotmail.com; 4 Department of Biochemistry, Faculty of Medicine, Ondokuz Mayis University, 55270 Samsun, Turkey; bahattinavci@hotmal.com

**Keywords:** coronary intervention, neuron-specific enolase, silent cerebral ischemia, stroke, transradial

## Abstract

*Background and Objectives*: Silent cerebral ischemia (SCI) is defined as a condition that can be detected by biochemical markers or cranial imaging methods but does not produce clinical symptom. This study aims both to compare the frequency of SCI in PCIs performed with right transradial access and left transradial access and to evaluate the influencing factors. *Materials and Methods*: A prospective, single-center study included 197 patients undergoing PCI via transradial access between November 2020 and July 2022. The patients were categorized into right radial and left radial groups. Neuron-specific enolase (NSE) values were measured and recorded before and 18 h after the procedure. A post-procedure NSE level higher than 20 ng/dL was defined as SCI. *Results*: SCI occurred in 60 of the 197 patients. NSE elevation was observed in 37.4% (*n* = 37) of the right radial group and in 23.5% (*n* = 23) of the left radial group (*p* = 0.032). Patients with SCI had higher rates of smoking (*p* = 0.043), presence of subclavian tortuosity (*p* = 0.027), and HbA1c (*p* = 0.031). In the multivariate logistic regression analysis, the level of EF (ejection fraction) (OR: 0.958 95% CI 0.920–0.998, *p* = 0.039), right radial preference (OR: 2.104 95% CI 1.102–3.995 *p* = 0.023), and smoking (OR: 2.088 95% CI 1.105–3.944, *p* = 0.023) were observed as independent variables of NSE elevation. *Conclusions*: Our findings suggest that PCI via right radial access poses a greater risk of SCI compared to left radial access. Anatomical considerations and technical challenges associated with right radial procedures and factors such as smoking and low ejection fraction contribute to this elevated risk.

## 1. Introduction

Since balloon angioplasty was first used in the treatment of ischemic coronary artery disease in 1977 [1], the frequency of percutaneous coronary intervention (PCI) has increased significantly, leading to an increase in associated complications. Local complications related to the catheter entry site, such as pseudoaneurysm, arteriovenous fistula, and retroperitoneal bleeding, are among the most common complications of PCI [2,3]. Additionally, cerebrovascular events such as stroke, transient ischemic attack (TIA), or silent cerebral ischemia (SCI) caused by atheroma plaques in the aorta and catheter-related thrombi may occur [4,5]. While procedure-related complications are less common in patients who undergo PCI via radial access [2,3,6,7], one study showed a higher frequency of SCI in these patients [8]. SCI is defined as a condition that can be detected by biochemical markers such as neuron-specific enolase (NSE) or cranial imaging methods but does not produce clinical symptom. NSE is a cytoplasmic intracellular enzyme found in neurons and peripheral neuroendocrine cells, used as a biomarker in the diagnosis and treatment of cerebrovascular accidents and other neurological disorders. NSE levels increase 2–4 h after acute neuronal injury, can be detected in the blood after 12–18 h, and remain positive for approximately 3 days [9,10]. In this study, we aimed to investigate the effects of right radial or left radial access on the development of SCI in PCIs performed with transradial access and the characteristics of the procedure affecting SCI.

## 2. Materials and Methods

This prospective, single-center observational study consisted of 197 patients who underwent coronary angiography with transradial access and subsequently underwent PCI between November 2020 and July 2022. The patients underwent PCI with right radial and left radial access, respectively, and were divided into two groups. Patients were randomly assigned (1:1) to the right transradial group or left transradial group. A web-based system (Research Randomizer (Version 4.0) from http://www.randomizer.org/) generated random numbers for patient allocation. The inclusion criteria were as follows: patients diagnosed with acute coronary syndrome and chronic coronary syndrome, who had stent implantation in at least one coronary artery.

Exclusion criteria were as follows: neurological malignancy (1); neuroendocrine tumors (2); degenerative diseases such as multiple sclerosis (3), Parkinson’s (4), and dementia (5); baseline NSE elevation (6); obvious stroke during PCI (7); aorto-ostial coronary interventions (8); hemodynamic instability (9); ST-segment elevation myocardial infarction (10); atrial fibrillation (11); previous CABG (12); previous stroke (13); failure to perform successful radial puncture (14); patients requiring transfer to the contralateral radial artery or femoral artery (15); and chronic total occlusion as the target lesion (16).

Patients who met the inclusion criteria underwent detailed examination by a cardiologist, including medical history, physical examination, and neurological assessment. Age, gender, smoking status, height, weight, and BMI were recorded. Before the procedure, lipid profile, glycated hemoglobin, complete blood count, kidney function, liver function, and coagulation tests were performed. The glomerular filtration rate (GFR) was calculated using the Modification of Diet in Renal Disease (MDRD) formula. Hyperlipidemia was defined as having a blood LDL level > 160 mg/dL or using antihyperlipidemic drugs. After PCI, all patients underwent echocardiographic evaluation and the ejection fraction was calculated and recorded using the Simpson method. A detailed neurological examination was conducted before the procedure and at 0 and 12 h after the procedure, including cranial nerve, motor, and sensory examinations. Patients whose radial pulse could not be palpated or for whom puncture could not be performed successfully were excluded from the study, and the study continued with same-sided radial access in the next patient.

Radial artery punctures were performed by experienced cardiologists, and the hydrophilic sheath (Ares 6Fr 7 cm) was placed using the Seldinger technique. To prevent radial artery spasm, 100 mcg IV nitroglycerin + 2500 IU Unfractionated Heparin (UFH) was administered through the sheath. In patients scheduled for stent implantation after coronary angiography, additional UFH was administered to achieve a total dose of 70–100 IU/kg. All patients received a loading dose of acetylsalicylic acid and a P2Y12 inhibitor before the procedure.

Judkins 5Fr catheters (Expo^TM^, Boston Scientific, Marlborough, MA, USA) were used in the left radial group, while imaging was started with the Tiger 5Fr (OPTITORQUE Coronary Diagnostic Catheter, Terumo Interventional Systems, Somerset, NJ, USA) catheter for diagnostic angiography in the right radial group. In cases in which successful engagement could not be achieved with the Tiger catheter, Judkins 5Fr catheters (Boston Scientific) were used. For patients undergoing PCI, the guiding catheter was selected according to the operator’s preference and lesion characteristics. Total number of materials and number of catheters, stent length, contrast volume, subclavian tortuosity, and target vessel were recorded. The total number of materials was defined as the sum of stents, balloons, catheters, and wires used.

There is no defined and accepted classification for subclavian artery tortuosity. In our study, the presence of severe subclavian tortuosity was defined as the need for complex catheter manipulation to reach the aortic root, the requirement for wire replacement with hydrophilic guidewire, catheter replacement, and the presence of anatomy at hairpin or spiral morphology. Cases in which the aortic root could be reached without the need for additional manipulation or in which the aortic root could be reached with simple maneuvers such as breathing and rotating the catheter by less than 90° were also considered as the absence of severe tortuosity. Patients requiring transfer to the contralateral radial artery or femoral artery due to severe tortuosity were excluded from the study.

Blood samples were collected before and at 12 h after the procedure and centrifuged (Electro-mag M815 P) at 3000× *g* for 10 min. Serum samples were stored at −80 °C. NSE measurements were obtained using the double-antibody sandwich enzyme-linked immunosorbent assay method with the Human NSE ELISA kit (Sun-Red Bio Company, Cat No. 201-12-0938, Shanghai, China). The mean inter-assay coefficient of variation (CV) was <12%, and the intra-assay CV was <10%. SCI was defined as a post-procedure NSE level higher than 20 ng/dL in patients whose pre-procedure NSE levels were within the normal range. 

All data analyses were performed using the SPSS (version 26.0, SPSS Inc., Chicago, IL, USA) program. The normal distribution of the data was assessed using the Kolmogorov–Smirnov test. For statistical analyses, mean ± standard deviations were calculated for parametric data, and median (minimum–maximum) values were calculated for nonparametric data. The chi-square test and Fisher’s exact test were used for differences between categorical variables. The Mann–Whitney U test was used for differences between medians, and Student’s *t*-test was used for differences between means. Independent variables affecting the frequency of SCI were evaluated with univariate binary logistic regression. Variables with *p* < 0.1 in the univariate analysis were then subjected to multivariate analysis. The significance level accepted in the study was *p* < 0.05.

## 3. Results

After initial evaluation, 212 patients met the study criteria. Among them, 15 were excluded for the following reasons: one had an obvious stroke post-procedure, one had a transient ischemic attack, two developed cardiogenic shock during the procedure and subsequently required inotropes, two underwent unplanned aorta-ostial PCI, one patient was transferred to the contralateral radial artery due to severe tortuosity, one patient was transferred to the femoral artery due to severe RAS, and seven had elevated basal NSE levels. After excluding these patients, a total of 197 patients were included in the study. Stent implantation and TIMI 3 flow were successfully achieved in all patients included in the study. The majority of the patients were male (69.5%), with a mean age of 62 years (Table 1).

When comparing procedure characteristics, including the contrast volume used, procedure time, fluoroscopy time, total stent length, postdilatation, predilatation, and guiding catheter, no significant differences were observed between the groups. However, the number of catheters (2.8 ± 0.6 vs. 2 ± 0.4, *p* < 0.01) and total materials used (6.7 ± 1.5 vs. 6.2 ± 1.7, *p* = 0.012) were significantly lower in the right radial group. The target vessel was the left anterior descending artery in 72 (36.5%), right coronary artery in 52 (28.9%), and left circumflex artery in 52 (26.4%) patients. In total, PCI was performed on more than one coronary artery in 14 (7.6%) patients. The target vessel distribution was similar between the groups (*p* = 0.772). Severe subclavian tortuosity was more common in the right radial group (6.1% vs. 23.2% *p* < 0.001) (Table 2).

In total, 60 patients had NSE elevations that met SCI diagnostic criteria. The patients were grouped according to SCI status, with NSE elevation observed in 37.4% (*n* = 37) of the right radial group and 23.5% (*n* = 23) of the left radial group (*p* = 0.032). The BMI was lower in patients with SCI (27.6 ± 3 vs. 29.10 ± 4.1, *p* = 0.033), and high HbA1c levels (*p* = 0.031), smoking (*p* = 0.043), and severe subclavian tortuosity (*p* = 0.037) were significantly higher in patients with SCI (Table 3). The factors affecting the frequency of SCI were evaluated using logistic regression analysis (Table 4).

Multivariate logistic regression analysis demonstrated that patients who underwent coronary procedures through the right transradial approach were more likely to develop SCI than patients with left transradial approach (OR: 2.104 95% GA 1.102–3.995 *p* = 0.023). Smoking (OR: 2.088, 95% CI: 1.105–3.944, *p* = 0.023) and left ventricular ejection fraction (OR: 0.958, 95% CI: 0.920–0.998, *p* = 0.039) were identified as other independent risk factors for SCI (Table 5). In ROC analysis, the sensitivity and specificity of LVEF for predicting SCI were 40% and 73%, respectively, and the cut-off value was 51.5% (*p* = 0,007; area under the ROC curve (AUC) = 0.639; 95% CI = 0.551–0.809) (Table 6).

## 4. Discussion

This study demonstrates that performing PCI via the right radial access, smoking, and low left ventricular ejection fraction values increase the risk of developing SCI. Periprocedural stroke occurs in a wide range of patients undergoing diagnostic coronary angiography (CA) and PCI, with reported rates of stroke/TIA of 0.05–0.1% in diagnostic CA and 0.18–0.44% in PCI procedures [11]. Both NSE levels and MRIs have similar sensitivities for diagnosing SCI [12], with studies reporting SCI in 11–42% of patients after CA and PCI [13,14]. While no significant difference was found between radial and femoral approaches regarding stroke/TIA in previous meta-analyses [15], radial access has been associated with a higher risk of SCI compared to femoral access [5]. To the best of our knowledge, this is the first study comparing the risk of SCI between right and left radial groups.

Neurological complications of CA include contrast-associated encephalopathy (CIE), ischemic stroke, and hemorrhagic stroke. Catheter manipulations within the aorta during PCI, physical removal and embolization of atherosclerotic material in the aortic wall, and other foreign objects and support devices used in the procedure may be causes of ischemic stroke. It may also present as a hemorrhagic stroke due to the antiplatelet and anticoagulant treatments administered during the procedure. CIE can present with visual impairment, focal motor and sensory loss, encephalopathy, seizures, global aphasia, and ophthalmoplegia. In addition to all these reasons, air embolism, micro-emboli, vasospasm, and intimal dissections can all be a cause of stroke.

Multivariate logistic regression analysis of the data showed that radial preference was a strong independent variable in predicting SCI risk. When examining subclavian anatomy, the right common carotid artery (CCA) was separated from the brachiocephalic trunk, while the left CCA was separated from the aorta by a different ostium. In right radial procedures, advancing materials such as catheters and wires through the subclavian anatomy may facilitate embolization of microthrombus and atherosclerotic plaques due to the proximity of the right CCA in their route. Additionally, our study showed that severe subclavian tortuosity is an important risk factor for SCI. In the presence of severe subclavian tortuosity, technical difficulties may arise in advancing materials into the coronary artery ostia. Complex and harsh maneuvers may be needed in situations that cannot be solved with standard maneuvers [16,17]. Denghani et al. reported that subclavian tortuosity was the main cause of failure and complications of right radial access procedures [18]. Severe subclavian tortuosity was more common in the right radial group, potentially contributing to SCI development in this group.

There are studies showing that heart failure patients with low (<40%) and intermediate (41–49%) ejection fraction (EF) are at higher risk for SCI [18,19,20,21]. Aykan et al. also supported in their research that low EF is a risk factor for SCI in patients undergoing PCI [8]. Left ventricular (LV) dysfunction has prothrombotic effects with endothelial dysfunction and platelet dysfunction. In multivariate logistic regression analysis of the data, the EF value was observed as an independent risk factor for SCI.

Cigarette smoking may cause ischemic brain edema by contributing to the disruption of the blood–brain barrier and affecting ion permeability due to the nicotine content [22]. Additionally, impaired ion permeability mediates neuronal death by increasing intracellular calcium levels and mitochondrial damage, potentially exacerbating ischemic brain injury [23,24]. Studies have shown that smoking is an independent risk factor for SCI, consistent with our findings [25,26].

Kotani K. et al. suggested that low BMI is a risk factor for SCI, particularly in patients > 81 years of age [27]. In another study, serum NSE levels were inversely related to BMI, and serum NSE levels decreased at a BMI > 25 kg/m^2^ [28]. This result may be related to the fact that excessive body fat negatively affects the brain structures in people with high BMI, reducing the gray matter volume and indirectly decreasing the serum NSE level in the body.

Studies have shown that diabetes is an important risk factor for SCI, but these studies do not include data for HbA1c [29,30]. Uncontrolled diabetes creates a predisposition to atherosclerotic changes with increased endothelial damage and vascular inflammation and causes the formation of aortic atheroma plaques. Plaque on the aortic wall may embolize due to wire and catheter manipulations during PCI, causing SCI. Additionally, diabetes causes microthrombus formation through platelet dysfunction and hypercoagulability. Our study showed that elevated HbA1c levels due to uncontrolled diabetes increase the risk of SCI, while the mere presence of diabetes is not a strong predictor of SCI development.

Our study needs to be evaluated while taking into account some limitations. Although the use of NSE for the diagnosis of SCI has been proven effectively in many studies, confirming the diagnosis with MRI and neuropsychiatric tests provides more reliable results. Additionally, all patients included in the study had severe CAD, so the risk of SCI could be more predicted. Another limitation of our study is that it was a single-center study with a relatively small sample size.

## 5. Conclusions

PCI performed via right radial access is associated with a higher incidence of SCI. These results highlight the need for careful consideration of the radial access side and risk factor management to reduce neurological complications in PCI procedures.

## Figures and Tables

**Table 1:** Comparison of baseline characteristics of left radial and right radial groups.

Parameter	Left Radial Group (*n* = 98)	Right Radial Group (*n* = 99)	*p*
Age (year)	61.4 ± 8.9 8.99	62.7 ± 8.6 8.65	0.307
Male, *n* (%)	69 (70.4)	68 (68.7)	0.867
Smoking, *n* (%)	49 (49.2)	43 (44.8)	0.393
Body mass index (kg/m^2^)	28.7 ± 3.9 3.92	28.5 ± 3.9 3.93	0.666
Hypertension *n* (%)	77 (78.6)	81 (81.8)	0.596
Diabetes *n* (%)	43 (43.9)	52 (52.5)	0.255
Hyperlipidemia *n* (%)	54 (55.1)	52 (52.5)	0.776
Prior MI *n* (%)	21 (21.4)	23 (23.5)	0.864
LVEF, %	54.8 ± 7.7	54.5 ± 7.4	0.803
Diagnosis at admission			0.762
NSTE-ACS *n* (%)	33 (33.7)	31 (31.3)	
CCS *n* %	65 (66.3)	68 (68.7)	
Hemoglobin, g/dL	13.7 ± 1.6	13.4 ± 1.7	0.252
Hematocrit, %	40.2 ± 4.4	39.3 ± 4.4	0.150
GFR, mL/dk	84.8 ± 22.6	80.1 ± 22.9	0.206
HbA1c, mg/dL	6.8 ± 1.6	7 ± 1.6	0.077
Triglyceride, mg/dL	185.2 ± 85.2	196.4 ± 117.5	0.529
Total Cholesterol, mg/dL	185.3 ± 45.6	188 ± 41.1	0.417
HDL-Cholesterol, mg/dL	41.0 ± 9.3	41.8 ± 10.4	0.586
LDL-Cholesterol, mg/dL	108.4 ± 39.5	110.3 ± 40.9	0.712

CCS, chronic coronary syndrome; GFR, glomerular filtration rate; HbA1c, glycated hemoglobin; HDL, high-density lipoprotein; LDL, low-density lipoprotein; LVEF, left ventricular ejection fraction; MI, myocardial infarction; NSTE-ACS, non-ST-segment elevation acute coronary syndrome.

**Table 2:** Comparison of procedural characteristics, P2Y12 status, and subclavian tortuosity of left radial and right radial groups.

Parameter	Left Radial Group (*n* = 98)	Right Radial Group (*n* = 99)	*p*
Contrast volume (mL)	154.8 ± 47.6	161.5 ± 50.8	0.276
Procedure time (min)	29.2 ± 11.7	31.4 ± 12.2	0.125
Fluoroscopy time (min)	14.1 ± 6.8	14.4 ± 7.3	0.980
Number of catheter, *n*	2.8 ± 0.6	2 ± 0.4	<0.001
Total number of used materials, *n*	6.7 ± 1.5	6.2 ± 1.7	0.012
Total stent length (mm)	29.6 ± 13.6	29.3 ± 14.0	0.805
Predilatation, *n* (%)	71 (72.4)	73 (73.7)	0.873
Postdilatation, *n* (%)	52 (53.1)	65 (65.7)	0.083
Catheter type			0.542
Judkins, *n* (%)	33 (33.7)	29 (29.3)	
Non-Judkins, *n* (%)	65 (66.3)	70 (70.7)	
SYNTAX score	11.5 ± 6.7	10.4 ± 5.7	0.388
Target vessel			0.772
LAD, *n* (%)	38 (38.7)	35 (35.4)	
LCX, *n* (%)	25 (25.5)	27 (27.3)	
RCA, *n* (%)	28 (28.6)	29 (29.3)	
LAD + LCX, *n* (%)	3 (3.1)	4 (4)	
LAD + RCA, *n* (%)	1 (1)	3 (3)	
RCA + LCX, *n* (%)	3 (3.1)	1 (1)	
Subclavian tortuosity			<0.001
Severe tortuosity (+), *n* (%)	6 (6.1)	23 (23.2)	
Severe tortuosity (−), *n* (%)	92 (93.9)	76 (76.9)	
P2Y12			0.955
Clopidogrel, *n* (%)	74 (75.5)	75 (75.8)	
Ticagrelor, *n* (%)	8 (8.2)	9 (9.1)	
Prasugrel, *n* (%)	16 (16.3)	15 (15.2)	

LAD, left anterior descending artery; LCX, left circumflex artery; RCA, right coronary artery.

**Table 3:** Comparison of baseline and procedural characteristics of patients with and without SCI.

Parameter	SCI (−) (*n* = 137)	SCI (+) (*n* = 60)	*p*
Radial preference			0.034
Left radial, *n* (%)	75 (76.5)	23 (23.5)	
Right radial, *n* (%)	62 (62.6)	37 (37.4)	
Age (year)	62.2 ± 8.6	61.8 ± 9.2	0.790
Male, (*n*) %	(90) 65.7	(47) 78.3	0.076
Body mass index (kg/m^2^)	29.1 ± 4.1	27.6 ± 3.0	0.033
Hypertension, *n* (%)	111 (81)	47 (78.3)	0.699
Diabetes, *n* (%)	61 (44.5)	34 (56.7)	0.117
HbA1c, mg/dL	6.7 ± 1.5	7.2 ± 1.7	0.031
Prior MI, *n* (%)	30 (21.9)	14 (23.3)	0.824
Smoking, *n* (%)	57 (41.6)	35 (58.3)	0.043
Hyperlipidemia *n* (%)	70 (51.1)	36 (60)	0.249
Subclavian tortuosity, *n* (%)	15 (10.9)	14 (23.3)	0.027
LVEF, %	55.4 ± 7.1	52.9 ± 8.2	0.061
Contrast volume (mL)	158.9 ± 50.5	155.3 ± 47.1	0.762
Procedure time (min)	29.9 ± 11.8	31.1 ± 12.5	0.506
Total stent length (mm)	30.4 ± 14.2	26.9 ± 12.5	0.108
Total number of used materials, *n*	6.5 ± 1.7	6.3 ± 1.5	0.552

HbA1c, glycated hemoglobin; LVEF, left ventricular ejection fraction; MI, myocardial infarction; SCI, silent cerebral ischemia.

**Table 4:** Predictors of SCI in univariate logistic regression analysis.

Parameter	Odds Ratio (OR)	%95 C.I. for OR		*p*
		Lower	Upper	
Right radial preference	1.946	1.047–3.676		0.035
Smoking	0.713	0.524–0.970		0.031
HbA1c	1.195	0.997–1.432		0.051
Male	0.530	0.261–1.075		0.079
Procedure time	1.009	0.984–1.034		0.505
Subclavian tortuosity	0.341	0.153–0.763		0.009
Total number of used materials	0.924	0.766–1.114		0.406
LVEF	0.958	0.922–0.966		0.033

HbA1c, glycated hemoglobin; LVEF, left ventricular ejection fraction.

**Table 5:** Predictors of SCI in multivariate logistic regression analysis.

Parameter	Odds Ratio (OR)	95% C.I. for OR		*p*
		Lower	Upper	
Right radial preference	2.104	1.102–3.995		0.023
Smoking	2.088	1.105–3.944		0.023
LVEF	0.958	0.920–0.998		0.039

LVEF, left ventricular ejection fraction.

**Table 6:** Area under the ROC curve (AUC) sensitivity and specificity by the optimized cutoff point for LVEF.

Risk Factor	AUC (95% C.I.)	*p*	Cut-Off Value	Sensitivity (%)	Specificity (%)
LVEF	0.639 (0.551–0.809)	0.007	51.5	40	73

LVEF, left ventricular ejection fraction.

## Data Availability

The data presented in this study are available on request from the corresponding author.
